# The Origin of Niches and Species in the Bacterial World

**DOI:** 10.3389/fmicb.2021.657986

**Published:** 2021-03-17

**Authors:** Fernando Baquero, Teresa M. Coque, Juan Carlos Galán, Jose L. Martinez

**Affiliations:** ^1^Division of Biology and Evolution of Microorganisms, Department of Microbiology, Ramón y Cajal Institute for Health Research (IRYCIS), Ramón y Cajal University Hospital, Madrid, Spain; ^2^National Center for Biotechnology (CNB-CSIC), Madrid, Spain

**Keywords:** bacterial niches, bacterial species, speciation, evolution, nichification

## Abstract

Niches are spaces for the biological units of selection, from cells to complex communities. In a broad sense, “species” are biological units of individuation. Niches do not exist without individual organisms, and every organism has a niche. We use “niche” in the Hutchinsonian sense as an abstraction of a multidimensional environmental space characterized by a variety of conditions, both biotic and abiotic, whose quantitative ranges determine the positive or negative growth rates of the microbial individual, typically a species, but also parts of the communities of species contained in this space. Microbial organisms (“species”) constantly diversify, and such diversification (radiation) depends on the possibility of opening up unexploited or insufficiently exploited niches. Niche exploitation frequently implies “niche construction,” as the colonized niche evolves with time, giving rise to new potential subniches, thereby influencing the selection of a series of new variants in the progeny. The evolution of niches and organisms is the result of reciprocal interacting processes that form a single unified process. Centrifugal microbial diversification expands the limits of the species’ niches while a centripetal or cohesive process occurs simultaneously, mediated by horizontal gene transfers and recombinatorial events, condensing all of the information recovered during the diversifying specialization into “novel organisms” (possible future species), thereby creating a more complex niche, where the selfishness of the new organism(s) establishes a “homeostatic power” limiting the niche’s variation. Once the niche’s full carrying capacity has been reached, reproductive isolation occurs, as no foreign organisms can outcompete the established population/community, thereby facilitating speciation. In the case of individualization-speciation of the microbiota, its contribution to the animal’ gut structure is a type of “niche construction,” the result of crosstalk between the niche (host) and microorganism(s). Lastly, there is a parallelism between the hierarchy of niches and that of microbial individuals. The increasing anthropogenic effects on the biosphere (such as globalization) might reduce the diversity of niches and bacterial individuals, with the potential emergence of highly transmissible multispecialists (which are eventually deleterious) resulting from the homogenization of the microbiosphere, a possibility that should be explored and prevented.

## Introduction

Niches are spaces for the biological units of selection. This opinionated review intends to address the origin, development, and evolution of niches from the viewpoint of ecology, as the uterine apparatus leading to the delivery of microbial individuals (units of selection), from species to stable communities in the bacterial world. The existence of a uterus has little meaning without the organism developing within. Similarly, the niche has no meaning without the organisms developing within, given that the concept of niches in a pre-biotic world is meaningless. It is the living individual organism that provides the niche’s identity. For example, the various rocky islands of the Galapagos were not “niches” for various species of finches *before* the stochastic arrival of the ancestral (undifferentiated) finches from the continent millions of years ago. They become “niches” because of the finches’ speciation. Once there is correspondence between a species and a niche, the niche perpetuates as such so that even if all members of the species go locally extinct, the empty niche would recognize or be recognized by a new incoming wave of individuals of the same niche-specific species, attracted by this area of historical accessibility. In fact, the origin of the word “niche” is related to the Latin “*nidus*,” a nest, providing conditions favoring the development of an organism. The bird builds its nest, which is essential for the species’ development. Eventually, another (probably related) bird species might recognize an alien nest as suitable for its own development (nest-robbing birds). Note that the nest (niche) construction frequently requires the branches of a particular tree in a particular environment (usually “historical,” i.e., one that has been used by previous generations) that can provide food to the nestlings, acting as a “nest” for the nest, thereby creating the intuitive image of a hierarchy of nests. How does this apply to bacterial speciation? Paraphrasing Jacques Monod from an anti-reductionistic perspective, “what’s true for birds is also true for bacteria.” We can certainly expand this consideration, recalling the fractal structure of nature, considering that “species” is a biological unity of individuation (building-up, developing the individual) and selection, and the niche (the nest) is the corresponding spatial physicochemical unit of individuation and selection. In fact, before the 16th century, the meaning of the term “species” was a “distinct class (of something) based on common characteristics,” that is, a category of something that can be appreciated (*specere*, “to see”) as an individual entity. The modern (biological) term of species was coined by John Ray in the 17th and extensively applied by Carl Linnaeus in the 18th century, referring to plants and animals. In our days, a certain resurrection of the old meaning is required, allowing to encompass biological individual entities below and beyond the classical definition of species.

The concept of bacterial species is particularly elusive. As with nest-robbing birds, several types of kin bacterial organisms fit in the same niche. It has therefore been proposed that the “niche” delineates the border of the microbial ecological units, not necessarily species but ecologically equivalent niche-specific organisms, “ecotypes,” so that the named species, composed by ecotypes, could resemble to what we now understand as a genus (Cohan, 2002; Ward et al., 2006). Indeed, the components of bacterial species are phylogenetically related and share a similar eco-phenotype (Rosselló-Mora and Amann, 2001). However, that attributed in this review to “species” could be equally attributed to any other evolutionary individual at higher (genus or even integrated communities of different species) and lower hierarchical levels (subspecies, clones) according to the different concepts of species (Palmer et al., 2019). The renewed interest in “niches microbiology” is fostered by the increasing need for determining the risks associated with the increasing anthropogenic effects on the microbiosphere. In the One Health and Global health perspectives, the human health, and the health of most of terrestrial life, depends on complex equilibria dominated by the conglomerating, integrative effects of microbiosphere. Such effects encompass from sustaining life of primary producers to the spread of resistance to antimicrobial agents (Baquero et al., 2019).

## The Concept of Bacterial Niche

The term “niche” was coined in 1913 by Grinnell and Swarth when discussing the speciation of Galapagos finches (Grinell and Swarth, 1913). and was conceived as a home for a single species or eventually subspecies. We should be aware that the term “niche” reflects an abstraction, in a sense a metaphor, and consequently attempts to a precise definition depends on the viewpoint and the intention of the scientist, which has resulted in an overall lack of clarity when was applied for different purposes. In our days, niche is simultaneously a space (but a fluid space, not a bubble), a scene where certain environmental abiotic, non-interactive and eventually variable conditions occur, an area where biotic-originated functions, including biological interactions at different hierarchical scales constantly modify the local conditions (as consuming or producing nutrients), and a flexible location where some organisms are settled (survive, reproduce) and other organisms migrate to accessible regions. Grinellian niches refer to multidimensional spaces shaped by an ensemble of independent, not or slowly interactive and non-consumable abiotic variables influencing organisms (scenopoetic variables, meaning not-interacting ones). Eltonian niches refer to local ensembles of fluid biotic interactions at cellular scale, including relatively static bionomic variables as competition for nutrients or mutualism (Soberón, 2007; Soberón and Nakamura, 2009). Note that all these different views were mostly born to approach biogeographical problems, including identification of distributional areas (frequently concerning plants and animals), but in fact they cannot be considered mutually exclusive. How these heuristic notions can be applied to microbial niches, which are widespread, but also fine-grained and discontinuous?

For the purpose of this review, focused on microbial niches, the meaning of niche is mostly based on that proposed by George Evelyn Hutchinson (1903–1991) (Hutchinson, 1957; Holt, 2009) as an abstraction of a multidimensional environmental space that can be characterized by a variety of conditions, both biotic (as bacterial interactions) and abiotic (the biotope), whose quantitative ranges determine the positive or negative growth rates of the evolutionary unit (typically a species but also communities of species) contained in that space. The Hutchinsonian concept of niche and biotope remains extremely useful in biogeography, ecology, epidemiology, and evolution but can be used to simulate the effects on microbial species and communities during niche formation and evolution under predicted local and global environmental changes (Colwell and Rangel, 2009).

The conditions of the Hutchinsonian niche recall an organism’s “phenotype,” and the traits of the “niche phenotype” should correspond (as in a “mirror-metaphor”) to those of the organism able to persist or grow within the niche. More than 25 years ago, one of the authors of this review (FB) termed the non-separable ensemble of these links the “ecobiome” (Baquero, 1994) expanding the first definition of “ecobiome” as the ensemble of biotic and abiotic conditions of an ecological niche (Polunin, 1984). The essential concept is the “niche response surface” derived from the population biology function *r(e)*, which is defined as the dependence of the population growth rate (*r*) on the ensemble of biotic and abiotic components of the environment (*e*) (Holt, 2020). Therefore, a niche is simultaneously an environment and a biological individual membrane, constituting an indissoluble ontological duality. The niche is “found” (opened) by the microbial variant. A particular environment where selection of a specific variant takes place is converted into a “niche,” which then individualizes (speciates, in a broad sense) the organism.

## Niches: Structure and Variation

The niche has an internal composition responsible for the “niche response” (Maguire, 1973), given that it is composed of a multidimensional ensemble of local conditions that are heterogeneously distributed in the niche, frequently forming gradients. In the niche centrality hypothesis, there should be a theoretical geometric center (“centroid” or “core”), representing the average overall quality of the niche. Within the niche, there should be a site where the combination of certain specific conditions is optimal for providing a hook to an introduced rare inoculum (propagulum) of a particular microorganism, so that it can persist and grow, ideally at or near the niche’s core. Hypothetically, the abundance of the niche-adapted species at this site should be maximal (Yañez et al., 2020). This situation recalls the Sewall Wright fitness landscapes, with optimal peaks where genotypes are abundant (Wright, 1932). As in the Wright landscapes, the core of niches does not need to be regular (a circle) and can be ovoid or even asymmetrical (Holt, 2020), with no single peaks but instead high ridges, given that one or more of the conditions in the niche can be more stable or dominant than the others in providing the maximum possible abundance (Holt, 2020; Osorio-Olvera et al., 2020).

However, the conditions that configure the core of niches might be unstable, which makes it difficult to identify the area of maximum abundance of a particular organism. The core therefore follows a trajectory than can be represented more as a geometrical locus than as a centrum. Microbial populations are expected to travel constantly in search of their fundamental core niche (or closer ones), and this co-variation, mediated by transmission events, mitigates the breaking of linkages between species and environments. In unstable environments, the abundance should be measured as the average densities along the geometrical locus and time. The niche is delimitated by an exclusive “membrane” that facilitates the organism’s reproductive isolation.

The movement (not infrequently oscillation) of the geometrical centroid of the original niche under varying conditions changes the niche’s parametric topology, which results in the opening of spaces with low abundance (peripheral niche) to eventual microbial variants able to deal with or exploit these suboptimal conditions, converting them into neo-cores for the variant genotypes and facilitating their permanence and growth. Although this situation should create clonal competition with the pioneer ancestor organism, the variability of the niche ensures coexistence and increases the possibility for the species (as an ensemble of microbial variants) to reach the niche’s full carrying capacity.

Niches with differing levels of complexity and stability clearly affect the evolution of bacterial populations. A “flat niche” with few components, strong dominance of one or several conditions, few and gentle gradients (Colwell and Rangel, 2009) and stable conditions across the niche should have a different effect on microbial species evolution than other niches. For instance, if the temperature (or osmolarity) is very high, the niche tends to be flat, as this component creates a single (or dominant) selective force. If the niche is both flat and large, the selection of microbial variants (and the resulting diversification and speciation) should decrease. In contrast, niches with high abundance and complexity but extremely efficient homeostasis (e.g., the cytoplasm of a eukaryotic cell) ensure a similar “flatness,” given that homeostasis coordinates/simplifies the components’ diversity, resulting in almost a single component with selective efficiency.

## Niche Stability and Adaptive Radiation

Sufficient “average stability” of the core niche-organism’s fidelity is critical for the speciation process. If the niche’s variation favors diversification of the species as an ensemble of subspecific variants, the speciation process requires “compartments” (stable nests) facilitating the organism’s reproductive isolation. Niche stability also ensures effective replication and the possibility of exploring stable neighboring environments to be converted into niches. This process of exploration, discovery, and colonization of neo-niches by phenotypic variants resulting in a rapidly growing lineage is termed “adaptive radiation” (Gavrilets and Vose, 2005; Gillespie and Parent, 2014). Paul Rainey et al. performed the seminal experiment demonstrating the emergence of new lineages (potential species) in stable environments (Rainey and Travisano, 1998). In a flask containing a shaken liquid culture, the inoculum of a bacterial type apparently remained genetically and ecologically homogeneous. However, if an inoculum is seeded into the flask without shaking, a compartmentalization of the culture’s conditions spontaneously occurs, creating spatially structured sub-environments [for instance, low oxygen content at the bottom, abundance on the surface, with particular air-culture-glass surface interactions (Jerdan et al., 2019)]. In a short period (days), “specialist genotypes” (in the culture plates) arise and preferentially occupy the various compartments when inoculated in a new flask. The genotypes are easily recognized by the particularities of the colonies they form when growing in agar plates. Several mutations have been found responsible for this adaptive radiation process, some more effective than others (McDonald et al., 2009). Adaptive radiation has also been observed in constant environment models (Maharjan et al., 2006), but it is difficult to rule out the presence of “microcompartments” in apparently unstructured stable environments, particularly if complex devices (such as a chemostat) are used.

The capacity for bacterial divergence without the need of ecological specialization in multiple niches was studied with a chemostat culture of *Escherichia coli* with limited nutrient resources (Maharjan et al., 2006). The originally clonal population radiated into several clusters with increased use of nutrients, competing but sharing the same niche (no periodic selection), and thus were considered to belong to a “single ecotype.” The multidirectional exploration of the fitness space is an underestimated factor in bacterial diversification. These *in vitro* studies have a correspondence with real life, and the sympatric speciation in the marine *Vibrio* genus is based on such early diversification in water microniches (Shapiro et al., 2012; Friedman et al., 2013).

## Niche Construction

The niche is an environmental template for microbial organisms, however, this individual (clone, species, integrated community of species) can modify the niche’s primary conditions, for instance, by consuming space and nutrient resources, altering the chemical composition as a consequence of metabolic and catabolic pathways, releasing bioactive macromolecules, causing horizontal gene transfer events, or providing structural surfaces, such as biofilms. In Rainey’s experiment described earlier, certain evolved “specialized genotypes” occur by exopolysaccharide secretion, contributing to the further formation of neo-niches (Lind et al., 2017). Niche construction is the process by which the niche is changed to suit the organism (Laland et al., 1999; Day et al., 2003), The activities of the organisms are “converted in niche,” so that the niche’s composition evolves over time and thereby influences the selection of a series of new variants in the progeny, so that the evolution of niches and organisms is the result of reciprocal interacting processes, which in fact constitute a single unified process (Woese, 2002). This process also occurs for communities of organisms, whose variability (composition fluidity) is also responsive to the eco-evolutionary feedback resulting from the evolution of niches.

The novel and changing features of the external environment might, however, enrich the niche by providing novel conditions. Microorganisms will convert the new ecological ensemble into a larger niche, following the emergence of new microbial variants. The “niche constructors” are the new variant organisms colonizing the peripheral border of the niche, where most changes occur, and new environmental traits are encountered. The more differentiated parts of the ensemble (such as clones, species) ensure the maintenance of the less specific higher hierarchies (such as phyla) in changing environmental conditions (García-García et al., 2019). A key evolutionary issue is whether the niche tends to be “preserved” and therefore “selected” over other environmental configurations by its microbiological content. We can consider that living entities exert a selfish “homeostatic power” that limits their niches’ variation. Although niche preservation is a widely used strategy, niche construction can also involve striking changes in previous niches conditions that can even involve a dramatic displacement of the microorganisms inhabiting the former niches. The best example of this is the accumulation of oxygen that began 2.4 billion years ago (Johnston et al., 2009), which entirely changed the Earth’s biosphere. In summary, the construction and evolution of niches is based on the biological activities of the organisms contained therein, adapting to the environmental conditions present in the niche and the niche’s periphery.

## Bacterial Niche Construction in Higher Species

Is the evolution of multicellular organisms a result of bacterial niche construction? Surprisingly, the evolution of mammals living in different environments and with quite different feeding habitats has scarcely affected the predominant bacterial phyla in their intestinal microbiomes. Although there are a hundred or so recognized bacterial phyla in nature, the actual number is probably more than 10 times higher (Hug et al., 2016). Only a few of these [Bacteroidetes, Firmicutes (constituting 90% of the microbiota), Proteobacteria and Actinobacteria] are consistently represented in mammals (Nishida and Ochman, 2018), which suggests that the maintenance (small divergence) of an old complex ensemble of a few microbial phyla has prevailed across the evolution of mammals, which could be interpreted by the microbiome’s seminal contribution to the shape and function of the intestine in ensuring the microbiome’s basic homeostasis. As in the case of the sterile newborns, there is there a “founder effect” involving the neighbor bacterial populations from kin individuals. This core phyla microbiota in mammals can diverge depending on the mammals’ evolution and diversification (Moeller and Sanders, 2020), mostly at the lower taxonomic ranks where particular types of bacteria tend to be associated with mammalian lineages, which implies a host niche-microbiota coevolution, probably based on differences in the intestinal chemosphere, including adaptation to diet and the local immune response (Shapira, 2016; Nishida and Ochman, 2018; Koskella and Bergelson, 2020). The need for maintaining the basic intestinal microbial niche under differing diets might have influenced the enlargement (possible niche construction) of parts of the gut (as foregut fermenters) to ensure long-term microbe-food interaction (Ley et al., 2008a, b). The “preservation of the intestinal niche” by microbiota might expand to the protection of the entire host in which this niche is located. Microbiota are known to affect the host’s health and immunological responses (Round and Mazmanian, 2009). The perception of the key-role of microbiota in human physiology has coined the expression “microbiome as a human organ” (Baquero and Nombela, 2012). This ecological mutual fidelity, resulting from coevolutionary events, explains why of all those tested, animal environments are by far the “most selective” for particular bacterial taxa (Tamames et al., 2010; Koskella and Bergelson, 2020). However, what is really selected are the “functions” of the members of microbiota, where functionally equivalent species can be selected, so that the functional microbiota is more stable than the taxonomic microbiota (Gosalbes et al., 2012).

## Niche’s Hierarchical Organization

Almost a century ago, the correspondence of evolution and “speciation” of biological individuals and environments suggested the possibility of establishing an ecologic hierarchy of regions, life zones, faunal, and plants habitats, as “niches,” paralleling the systematic hierarchy from life kingdoms to subspecies (Grinell, 1928). An obvious corollary of the abovementioned “mirror metaphor” linking microbial individuals (from phyla to species and communities of species) and niches is that the taxonomic hierarchy of microbes should correspond to a hierarchy of niches, starting with those corresponding to the smaller microenvironments (Baquero, 2009). However, the full characterization of the traits defining microenvironments remains in its infancy (Baquero, 2015), although there has been significant methodological progress in recent years in this direction (Wessel et al., 2013; Aghajani Delavar and Wang, 2020). There are macroniches that are likely exploited by organisms of higher hierarchies, as well as microniches resulting from microenvironments, where the microorganisms expand and evolve.

Macroenvironments are composed of microenvironments, just as a photograph can be pixeled into smaller portions. Analogous microenvironments can likely be found in diverse macroenvironments. Each of these small parts cannot be recognized by the biological entities of higher hierarchies lacking the analytical tools to explore and exploit the qualities of potential small niches. Microenvironments are converted into microniches when they become specifically occupied by a particular bacterial species. The minimal niche should correspond to a specific clone or subclone, hypothetically to a single cell lineage. Microenvironments differ from macroenvironments by their higher number and diversity, essentially based on the differences and relatively higher local representation (concentration) of certain environmental components, thus providing specific qualities. Microenvironments are more influenced than macroenvironments by the effect of spatial-temporal physical and chemical gradients, in turn influencing the closer neighbor micropatches. As will be discussed next, gradients are of considerable interest in explaining diversification and speciation.

## Niches, Gradients, and Bacterial Diversification

The astonishingly high diversity of lifeforms, particularly in the lower taxonomical levels, suggests that microniches emerge along continuous environmental gradients. The possibility that particular microbial variants with differing selective specificity might emerge at different points in a concentration gradient was shown during the 1990s. The apparent continuum where quantities (chemical, physical) are converted into qualities (units of activity) able to select specific genotypes recapitulates Leibniz’s *Calculi Differentialis* for the evolutionary field (Baquero and Negri, 1997; Baquero et al., 1997; Negri et al., 2000). Given that the gradients are frequently unstable, the selected variant genotype might follow the concentration at which it was selected, provoking a continuous state of flow, which might facilitate exposure to other areas of the gradient or other crisscrossed selective gradients, resulting in the possibility of novel variations (with possible epistatic effects). The selection of a particular species or population in a segment of a gradient immediately influences the gradient itself (for instance consuming O_2_, or lowering the pH), or create new associated gradients, as gradients of nutrients, inhibitory substances, quorum-sensing molecules, or pheromones, contributing to the fluidity of the multidimensional niche landscape, and the intensity of biotic interactions. That facilitate genetic exchanges with neighboring kin populations sharing the same selective compartment, increasing the evolvability of the lineage (Chomicki et al., 2020).

## Diversification, Specialization, and Speciation

The law of universal differentiation, which has been termed “adaptive radiation,” has been present since the prehistory of evolutionary biology, with contributions from Lamarck, Huxley, Cope, and Darwin (Osborn, 1902). There is a spontaneous propensity for evolutionary individuals to differentiate, probably resulting from the sequential accumulation of random events, in accordance with the second law of thermodynamics, such that variation in bacterial genome sequences continuously increases (Wang et al., 2016). McShea and Brandon (2010) proposed that this tendency followed the “zero-force evolutionary law,” which states that “in any evolutionary system in which there is variation and heredity there is, in the absence of constraints, a random tendency for diversity and complexity to increase.” From this viewpoint, diversification might occur simply by neutral drift without the need for nichification. In fact, studies on the timetree diversification of bacterial organisms have shown an exponential increase in new lineages with no clear evidence of saturation, regardless of the constant net diversification rate derived from the niche’s emptiness or environmental variation (Marin et al., 2017).

Diversification is a centrifugal force, whereas speciation is a centripetal one. Processes as clonalization (subspecific genetic and ecological diversification) might be thought of as analogous to speciation, although that is not necessarily true. Speciation implies a diversification from an ancestor; however, diversification, even in combination with isolation, is insufficient for building a bacterial species. On the contrary, speciation might result from an anti-diversification process by the condensation of different niche specialists that evolve late in the adaptive radiation process, overcoming competition and adapting to the original different niches (Meyer et al., 2011), which might be considered a general feature of evolutionary biology defined as “*ex unibus plurum*” and “*ex pluribus unum*” dynamics (Baquero, 2011). Higher diversification rates during adaptive radiation occur during the early stages, a phenomenon that also occurs in ecological communities (Calcagno et al., 2017). At later stages, diversity tends to decrease, which might be an intrinsic consequence of diversification, a kind of overshooting dynamic (Meyer et al., 2011). Reversing the course of diversification is a rare event, given the “Müller’s ratchet.” In addition, diversified variants might break the bridges with the coexisting ancestor, facilitating isolation, sometimes using a direct “inhibition of the ancestor strategy” (Baquero and Lemonnier, 2009). Through convergent evolution, similar phenotypes might evolve among different lineages, and such convergence might reduce diversification when lineages have overlapping niches (Speed and Arbuckle, 2017). In principle, diversity reduction might be a consequence of increased competition among variants. However, it might also occur through the condensation of radiating genotypes in new environments (neo-niches) and hybridization (commonality) of the selected neighbor genotypes, resulting in the establishment of a “key innovation” leading to global adaptation, faster selection and increasing abundance. The consequence on niche dynamics is that after a period of increased niche diversity, a coalescence of niches (increasing niches’ breadth) might occur (Seehausen, 2004; Shapiro et al., 2012; González-Torres et al., 2019) which eventually results from the emergence of various advantageous mutations (MacLean and Bell, 2003) which can be followed by niche expansion.

Genetic exchange is common among bacteria, and has probably influenced old bacterial speciation (de la Cruz and Davies, 2000; Ochman et al., 2000; Brown, 2003), and genetic evolution and speciation of bacterial populations and “community species” might resemble that of recombinant sexual eukaryotes. However, its effect on population diversity during ecological differentiation remains controversial. A fundamental question is whether advantageous mutations lead to the selection of clonal genomes or, as in sexual eukaryotes, sweep through populations on their own. Ecological differentiation (akin to a sexual mechanism) has occurred in the recently diverged populations of ocean bacteria (*Vibrio*). A few genome regions have swept through subpopulations in a habitat-specific manner, followed by a gradual separation of gene pools and an increased niche specialization of the most recent recombinant populations (Shapiro et al., 2012). The possibility of microbial speciation by geographical isolation over a long-time scale was suggested for thermophilic archeobacterium found in self-igniting coal piles or in sulfurous hot springs, and in cyanobacteria from distant parts of the world (Papke et al., 2003; Whitaker et al., 2003; Cadillo-Quiroz et al., 2012). However, the generality of these observations is totally dependent of a precise definition of “species” (Papke et al., 2007).

## The Keys for Opening New Niches: Mutation, Recombination and Horizontal Gene Transfer

The exploration of and adaptation to possible new niches imply population diversification, which occurs by mutation or horizontal gene transfer. The success (fixation) of novel divergent subpopulations is further determined by the magnitude of the genetic changes and by the gene flow barriers that prevent population mixing and taxon convergence (Friedman et al., 2013; Palmer et al., 2019). Genetic changes occur at differing evolutionary rates, time scales (Diaz et al., 2019), and ecological proximities, giving rise to diversification-speciation based on sympatry (occurring without significant spatial separation), allopatry (in fully separate locations), or parapatry (separated, but maintaining contacts) (Whitaker, 2006; Friedman et al., 2013; Meyer et al., 2016; Chase et al., 2019). Accordingly, these changes involve differing “speciation/adaptive genes,” from housekeeping to niche-specific genes (Orr et al., 2004; Nosil and Schluter, 2011) and adaptive genes, allowing for rapid or intermittent environmental stresses (e.g., antibiotic resistance). Studying the natural diversification of a single bacterial lineage over time, as a result of “opening new niches” in an otherwise stable environment, was the goal of the seminal long-term evolution experiment launched in early 1988 by Richard Lenski in 12 parallel minimal medium cultures of *E. coli* that survived for more than 70,000 generations. The author detected mutational divergent evolution, with new niches occasionally opening up, such as the ability to use citrate as a single carbon source, an extremely unusual trait (which might have been lost or silenced) in this species, thereby likely preventing the colonization of certain habitats (Blount et al., 2008: Lenski, 2017). In this experiment, divergent evolution was also a consequence of large intrachromosomal rearrangements (deletions, inversions, duplications), probably mediated by insertion sequences (Raeside et al., 2014).

**FIGURE 1:**
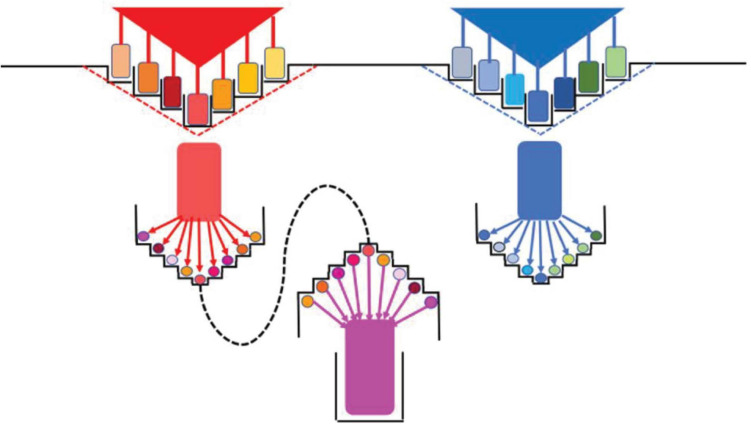
Niches, diversification, and commonality of bacterial populations. **Top:** Two ancestor populations (red and blue triangles) that were adapted to their primitive, fundamental niches (broken lines); at the sides, the stair-like profiles represent the subdivision of the biotope by gradient formation. Different spaces in the gradient facilitate the diversification of the ancestor population in variants (colored rectangles), splitting the ancestor niche into new specific niches (adaptive radiation). **Middle:** Each of these specific variants further pixelates the biotope, taking advantage of sub-gradients, resulting in new rounds of diversification (red colored circles) and nichification (new adaptive radiation). **Low:** There is a limit to the diversification process, when neighbor variants start to exchange adaptive information, eventually producing an adaptive commonality and the emergence of a higher entity population (species?).

Horizontal gene transfer is a major force in prokaryotic adaptation and diversification and might therefore foster niche coalescence, requiring a certain phylogenetic neighborhood and occurring more frequently among subspecies (Figure 1), thus overcoming the natural resistance to interspecies recombination in the core genome (Lefébure et al., 2010). However, there might be restrictions to large recombination events among lineages of the same species (Comandatore et al., 2019). Extensive recombination events in the core genome of lineages of a given species result in hybrid organisms known as “hopeful monsters” (Croucher and Klugman, 2014). The term “hopeful” refers to the possible emergence of a key innovation, i.e., an unexpected sudden adaptive success in exploiting a new niche, which frequently involves parapatric prokaryotic populations that occupy adjacent niches with intermittent gene flow. This type of large-scale recombination has been shown in species from *Vibrio*, *Klebsiella pneumoniae, Streptococcus*, and *Staphylococcus* (Coyle et al., 2020). Within the core genome, the integrated set of genes involved in the basic but highly complex process of conveying genetic information (such as transcription and translation) is less frequently transferred than other genes, which are expected to have been continuously acquired since the origin of Prokaryotes, as postulated by the complexity hypothesis (Jain et al., 1999).

Early processes of bacterial diversification in the search for and adaptation to new niches appear to be associated with the acquisition of “niche-specific traits” often located on mobile genetic elements (Touchon et al., 2020), which include a plethora of genes such as those involved in host or environmental adaptation (colonization, virulence, biodegradative pathways), often in large islands or operons (de la Cruz and Davies, 2000). Whether antibiotic resistance (or reducing damage through immunity) contributes to the creation of new “speciating” niches is a matter of interest. Antibiotics select certain lineages (those that have acquired the resistance trait) that might prevail even in the absence of antibiotic exposure. Spatial or temporal separation facilitates the fixing of these gene changes, thereby facilitating the diversification of species in relation to ancestral genotypes, which is especially evident in allopatric populations that face adaptation to different ecological niches.

Diversification occurs in sympatric populations occupying either non-overlapping niches and overlapping niches that are extremely frequent in nature (such as in water bodies, or and gut mucosa). Both mutations and mobile genetic elements maintain the heterogeneity and robustness of the necessary ensemble of bacterial populations to persist in changing environments (Heuer et al., 2008).

The co-existence of diverse genotypes of a given species in a given niche remains intriguing at the evolutionary level. This consistent species/strain heterogeneity observed in different geographical areas for certain opportunistic pathogens might reflect ephemeral genotypes or the sampling of different local transmission events or microepidemics, although migration and further population diversification, repeated disseminations and local species-wide extinctions also appear to be plausible explanations. The specific gene content of some of these multistrain pathogens is limited, revealing poor adaptation to particular niches but with the ability to repeatedly explore through transiently acquired mobile loci or mobile genetic elements (Harrow et al., 2021).

## Niche Variability, Core Genomes, and Accessory Genomes

The dynamics (stability, change) of macroniches, as those potentially associated by multicellular animals or plants, can be compared with microniches, the field of unicellular organisms, as bacteria. In both cases, the different niche periodicities should influence the evolutionary potentiality of the corresponding organisms; short periodicities (higher stability) are expected to occur in oscillatory systems. In principle, the parametric composition of macroniches should be more stable in time, and more transient in microniches (Brock, 2000). However, the variability of microbial niches is probably buffered by the extreme abundance of microniches, so that a lost niche does not necessarily involve the extinction of the associated organism or community. On the other hand, many microniches are in homeostatized systems (for instance inside animals, plants, tissues, or cells) assuring a high degree of stability.

The correspondence between niches and biological individuals should be reflected in the correspondence of the core of niches and the core of bacterial genomes. The core genome is the ensemble of genes that are shared by all members of the species and the accessory genome, which are present in only a certain proportion of the group members (Soucy et al., 2015). The sum of the core and accessory genome is the pangenome. The exact proportion of core genome per bacterial species is difficult to ascertain, because this proportion is highly dependent on sampling; the more genomes in the database (highly biased by research and epidemiological interests), the greater the expected detected proportion of the accessory genome (Rouli et al., 2015). However, the proportions of core and accessory genomes in pangenomes are extremely variable among species with different lifestyles, colonizing ubiquitous or extremely specific niches. Ubiquitous and relatively flat, stable niches, with a predominance of key-component or high homeostatization, reduces the need for acquiring new traits toward local specialization. Bacterial organisms that evolve in this type of niche tend to have a higher proportion of the core genome in the pangenome. The extreme genome core dominance in the pangenome occurs in symbiotic species with high host niche dependence and do not tolerate variation. *Buchnera aphidicola*, an insect endosymbiont, is an example of evolutionary genomic stasis, with a fully preserved genome over the past 50 million years (Tamas et al., 2002). Bacterial symbiont stasis is due to reductive evolution, reaching only 140 genes (Martínez-Cano et al., 2015). In highly stable environments, predominantly intracellular bacteria such as *Chlamydia* and *Rickettsia* also have a predominant core genome. Widespread species such as *Pseudomonas aeruginosa*, or certain *Bacillus*, but also *Listeria monocytogenes* or *Legionella pneumophila* (that also have intracellular niches), have a relevant core genome, adapted to widespread and/or homeostatized niches (Baquero et al., 2020).

Genes that are now recognized as a “core genome” in a given genus or species were not necessarily “core” in the ancient history of the lineage. Those genes that encode basic elements of the bacterial machinery (such as replication, transcription, and translation) are needed in all habitats; however, it is highly likely that many metabolic, regulatory, stress-response, and signaling core genes were “accessory” in this distant past. This means that the complexity of ancestor niches was either simpler, or at least relatively simpler, that is, dominated by a few powerful conditions, or were simply insufficiently dug up, exploited and far from their carrying capacity. The search for cores by using stratified phylogenies in successive supraspecific and subspecific taxons supports such a hypothesis (Gonzalez-Alba et al., 2019).

## Selfish or Cohesive Speciation

Species have boundaries that are frequently surrounded by a taxonomical empty space (or sparsely populated, “*quasi terra nullius*”), thereby shaping the individuation, discontinuity, granulation, and cluster-and-gap pattern observed in the biosphere. In the case of bacterial species, the whole genome’s average nucleotide identity (ANI) identifies as members of the same species those organisms showing ≥95% average nucleotide identity among themselves, and the taxonomical empty space can be extended until reaching less than 83% average nucleotide identity (Jain et al., 2018). The borders can be considered to correspond to the niche occupied by the species (Holt, 2009), or higher-level individual entities, and the first occupants (priority effect) “protect” (in a selfish, cohesive way) the ownership of the niche either by increasing the fitness by extracting nutrients from the periphery or by producing amensalistic substances to prevent foreign invasions (Cohan, 2019; Grainger et al., 2019), which naturally contributes to the increase in reproductive isolation: the classic Mayr’s condition for speciation (Mayr, 1942; De Queiroz, 2005).

## The Evolution of Niches

The evolution of niches and species constitutes a single process, as the linkage of niches and species occurs between “processually equivalent” entities (Bapteste and Dupré, 2013). As stated in the preceding paragraph, there is a common historical dimension of niches and species (Sykora, 1995). Niches came into existence with the emergence of life, but life contributed to the expansion and diversification of niches. The first colonizers were simple organisms, all core genomes, with the minimum set of functions to survive and replicate. With all the biases that affect the composition of databases, only approximately 200 genes constitute the Bacteria Domain core genome (Gil et al., 2004), which are common for all bacterial organisms. As a remnant of this ancestral core, approximately 50 genes are still retained in current *Escherichia coli* genomes (Gonzalez-Alba et al., 2019). Most of these genes correspond to the basic cell machinery involved in mRNA and protein synthesis/replication, with no genes that can provide clear insight into the ancestor niche. This indicates that this deep core genome is a bacterial precondition for niche exploitation, which in turn depends on the accretion of genes by introgression (Bapteste, 2014), either to protect the integrity of the essential deep core or to exploit novel environments and convert them into niches. Such a way, the history of the bacterial genome should illustrate about the changing composition of the niches that this organism has exploited from ancient times (Alcaraz et al., 2008). Research into the origin of accessory genes (in particular, the accessory genes of the smaller free-living prokaryotes, with approximately 800 genes) might reveal the structure of old niches, constituting a fertile field for future research (Croll and McDonald, 2012; Martínez-Cano et al., 2015), that should be based on a better understanding of geology and the environmental setting at the past, at the “lost world” (De Anda et al., 2018; Souza et al., 2018). The evolution of niche breadth (expansion or contraction) strongly corresponds to the generalist of specialist lifestyle of the species contained (Sexton et al., 2017).

It is obvious, however, that the physical, chemical, and nutritional conditions of microbial niches are influenced by abiotic events, frequently of stochastic nature, including those resulting from direct or indirect anthropogenic effects (such as chemical pollution and climate change). The stochastic variation in niches is the equivalent of mutational events; when this results in the merging of niches, it corresponds to recombination in the biological world. These changes, which alter the equilibrium, immediately become challenges for the biological part of the niche, fostering the adaptive genetic diversification of the pioneer organism, resulting in niche evolution (Holt, 2014).

## Niche Abundance

**FIGURE 2:**
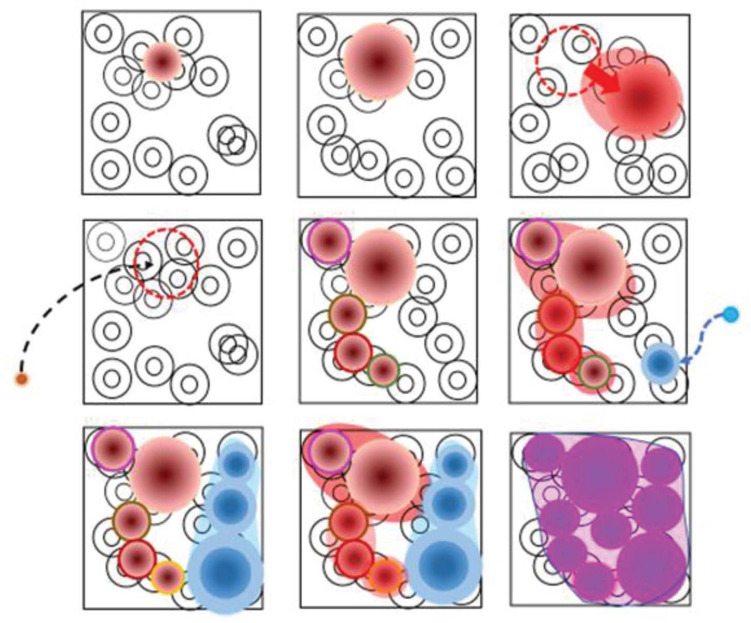
The process of conversion of biotopes in niches. The figure represents nine successive stages of nichification of a biotope, whose constituents are depicted by target-like circles, to represent gradients. **First row:** **(left)**, a small bacterial population finds a selective combination of traits able to sustain its growth (brown circle); the limits are those of the current niche; the color intensity reflects the bacterial abundance; **(middle)**, the population is expanding and converting in niche the neighboring regions of the biotope; **(right)**, the combination of traits defining the niche is shifting to the right, so that the niche moves into the biotope space. **Second row:** **(left)**, an extinction event has eliminated the pioneering colonizer population; however, from the outside environment, a new small population of the same organism enters the biotope, recognizes the conditions of the ancient niche, and reinstalls itself; **(middle)**, the sequential variants of the pioneering population emerge. This variation allows for the expansion of the niche; **(right)**, a new invader of the biotope (blue circle) produces a new niche. **Third row:** **(left)**, sequential variation of the new population expands the blue niche; **(middle)**, niches of the brown and blue populations start to converge; **(right)**, this convergence facilitates genetic exchanges between brown and blue populations, creating a new pink population (species?) advancing the exploitation of the biotope’s carrying capacity, which is almost entirely converted in a niche.

Niches are spatial combinations of environmental traits (biotopes) that are colonized (“nichified”) by microbial genotypes (Figure 2), some of them extremely ubiquitous. The interpretation of the Lourens Baas-Becking and Martinus Beijerinck hypothesis “Everything is everywhere, but the environment selects” (De Wit and Bouvier, 2006) should be nuanced because the environmental hot points for selection are not ubiquitous (everywhere). In principle, areas with a larger extension, where environmental diversity is expected to be higher, should provide an abundant supply of different types of niches. This abundance represents the “area effect” that affects the selection of genotypes and ensures larger populations, greater potential variation to expand the basic population in transmission events, and greater distance between niches, thus contributing to the genetic isolation and dilution of the locally deleterious genetic traits (Gavrilets and Vose, 2005).

## Empty Niches, Niche Reproduction, and Biological Transmission

Niches produce organisms, and organisms produce niches. A paradigmatic example is the newborn mammal’s intestine, an empty (sterile) niche at birth but able to recognize and host a complex community of microbial populations. The term “empty niche” is certainly an internal contradiction, but we used such expression to help to imagine a conserved (and reproducible) ensemble of anatomical and biochemical conditions, in a sense, a “potential niche.” The intestine’s anatomy is adequate for ensuring successful microbial colonization, and this empty niche is a “memory of an occupied niche.” In this sense, there is niche reproduction, ensuring the reproduction of a complex microbial system (Baquero, 2014). We can apply the same concept to numerous other primarily empty niches that are “reproduced,” particularly (but not exclusively) those that are built by plants and animals. Varying degrees of “emptiness” might occur by total or partial extinction of the biological individual(s) contained in the niche, occasionally by anthropogenic effects. Given that nature abhors a vacuum (in Grinnell’s words), niches tend to be re-occupied by microbial organisms that are kin (“ecologically equivalent”) to those that disappeared, until the niches’ maximum carrying capacity is reached. Microbial diversity is thereby maintained, as occurs with species higher up the taxonomical hierarchy (Walker and Valentine, 1984). The opposite of niche emptiness is niche overgrowth, when the niche’s carrying capacity is almost saturated and its space cannot be expanded, despite continuing replication and immigration. In this case, the important process of transmission occurs (Baquero, 2017, 2019), where organisms are compelled to migrate in search of compatible niches (Hugh Dingle and Ali Drake, 2007). The success of this migration process depends on the abundance of accessible niches in the area.

## Opportunistic Infections, Niche Deconstruction and Short-Sighted Evolution

Multicellular organisms in general and humankind in particular constitute a set of nutrient-rich environments able to support the growth of a large number of microorganisms (Levin and Antia, 2001). However, the host’s tissues do not contain microorganisms, unless infected, and the organisms’ surfaces are colonized by stable microbiomes that, together with anti-infection defenses, impede the entrance of other microorganisms. Typical pathogens can sometimes deal with these defenses by expressing virulence factors acquired through evolution. The colonization of the novel (empty) niche by expressing virulence has driven the evolution of such pathogens, the best studied example of which is the evolution of *Yersinia* (Wren, 2003). There is, however, another category of pathogens: those causing opportunistic infections, which can colonize the inner host niches without the prior acquisition of virulence determinants (Martínez, 2013). These opportunistic pathogens have historically included human commensals microorganisms and, since the introduction of antibiotics, antibiotic-resistant environmental bacteria. These organisms rarely produce infections in healthy individuals but can infect those with underlying diseases, extended injuries/burns, immune problems, debilitation and impaired microbiomes due to the potential niches offered by the fact that these hosts are less stringent than the equivalent niches in healthy hosts. In this case, niche shifting (from natural environments to infective ones) is the consequence of the host’s niche deconstruction, in the sense that certain characteristics that impede niche colonization by outside agents have disappeared or become more relaxed. The entrance into the new accessible niche, however, might foster the evolution of these opportunistic pathogens (Rossi et al., 2020). Although some might consider an end to this process, such as the case for classical pathogens that end in speciation, this does not seem to be the case. Evolution to colonize a new niche implies the de-adaptation from the former niche. In the case of regular pathogens that are able to infect any host-niche, the amount of interconnected hosts is sufficient to allow the evolved organism to persist in these niches (endemics, epidemics). In the case of opportunistic pathogens, however, the number of potential hosts is far more reduced. The microorganisms return to their original niche where they are outcompeted by their non-de-adapted, evolutionary progenitors (Sokurenko et al., 2006) in a process that has been termed “short sighted evolution” (Levin and Bull, 1994; Martínez, 2014).

## The Consequences of Anthropogenic Uniformization of Niches

The anthropogenic effects of bacterial niches should be explored more responsibly. These effects are essentially due to the direct alteration of the macrobiosphere (reduction in plant and animal biodiversity) and microbiosphere (reduction in biodiversity by toxic agents, such as antimicrobials, metals, and biocides). The disappearance of a microbial species might have significant consequences for the biosphere (particularly on the primary producers) and thus on global health (Hernando-Amado et al., 2019). Anthropogenic globalization influences not only socioeconomic aspects but also ecology, and ecology affects health. This process started with the activities of early humans, probably during the Neolithic period (Key et al., 2020). In recent centuries, however, the density and intensity of human activities have grown exponentially. Globalization facilitates the exchange and transmission of microbes worldwide, while simultaneously homogenizing habitats and potential niches, resulting in microbial stress and reductions in microbial diversity. A paradigmatic example is the expected reduction in the diversity of microbiotas in different hosts, facilitating their merging and coalescence (Baquero et al., 2019). Microbiotas are getting sick (Renson et al., 2020). It is currently difficult to predict whether these effects will accelerate or constraint bacterial diversification and speciation, but a larger number of common or overlapping niches might result in the emergence of highly generalized/transmissible new bacterial organisms, eventually potentially high-risk pathogens for higher plants and animals, including humans. The time has arrived to review the trends on microbiosphere diversity, given that we are creating new potential niches of unpredictable influence on the world’s equilibrium.

## Author Contributions

All authors listed have made a substantial, direct and intellectual contribution to the work, and approved it for publication.

## Conflict of Interest

The authors declare that the research was conducted in the absence of any commercial or financial relationships that could be construed as a potential conflict of interest.
